# Genotyping-by-Sequencing Defines Genetic Structure within the “Acquaviva” Red Onion Landrace

**DOI:** 10.3390/plants11182388

**Published:** 2022-09-13

**Authors:** Chiara Delvento, Stefano Pavan, Monica Marilena Miazzi, Angelo Raffaele Marcotrigiano, Francesca Ricciardi, Luigi Ricciardi, Concetta Lotti

**Affiliations:** 1 Department of Soil, Plant and Food Sciences, Section of Genetics and Plant Breeding, University of Bari Aldo Moro, Via Amendola 165/A, 70126 Bari, Italy; 2 Department of Agriculture, Food, Natural Resources and Engineering, University of Foggia, Via Napoli 25, 71122 Foggia, Italy

**Keywords:** onion, landrace, SNPs, genetic structure, traceability

## Abstract

Genetic structure and distinctive features of landraces, such as adaptability to local agro-ecosystems and specific qualitative profiles, can be substantially altered by the massive introduction of allochthonous germplasm. The landrace known as “Cipolla rossa di Acquaviva” (Acquaviva red onion, further referred to as ARO) is traditionally cultivated and propagated in a small area of the Apulia region (southern Italy). However, the recent rise of its market value and cultivation area is possibly causing genetic contamination with foreign propagating material. In this work, genotyping-by-sequencing (GBS) was used to characterize genetic variation of seven onion populations commercialized as ARO, as well as one population of the landrace “Montoro” (M), which is phenotypically similar, but originates from another cultivation area and displays different qualitative features. A panel of 5011 SNP markers was used to perform parametric and non-parametric genetic structure analyses, which supported the hypothesis of genetic contamination of germplasm commercialized as ARO with a gene pool including the M landrace. Four ARO populations formed a core genetic group, homogeneous and clearly distinct from the other ARO and M populations. Conversely, the remaining three ARO populations did not display significant differences with the M population. A set of private alleles for the ARO core genetic group was identified, indicating the possibility to trace the ARO landrace by means of a SNP-based molecular barcode. Overall, the results of this study provide a framework for further breeding activities and the traceability of the ARO landrace.

## 1. Introduction

Common or bulb onion (*Allium cepa* L. 2n = 2x = 16) is cultivated on about 5.6 million ha worldwide (FAOSTAT data, 2020 [1]) and widely used for human consumption. It is also one of the main ingredients of several traditional recipes, and a valuable source of nutrients, vitamins, minerals, and bioactive compounds [2,3,4,5,6]. Southwestern Asia is considered the onion domestication center, while several secondary diversification centers occur in the Mediterranean Basin [7].

Considerable genetic diversity is available in common onion landraces and cultivars that are cultivated in the field and/or preserved in ex situ collections established worldwide [8]. A few studies investigated the genetic variation of global diversity panels [9,10], whereas several works focused on the phenotypic and genetic characterization of landraces of main interest for local economies and breeding purposes (e.g., [11,12,13,14,15,16]).

The Italian onion production is significant at the global level (458 thousand tons/ha—FAOSTAT data, 2020 [1]) and is mostly referable to open-pollinated landraces, adapted to local agro-ecosystems and showing wide phenotypic variation with respect to shape, dormancy, bulb color, pungency, and nutritional features. The genetic diversity of these landraces was poorly investigated so far, thus limiting the possibility to protect them from genetic contaminants and frauds, as well as their exploitation in breeding programs [9].

**Figure 1:**
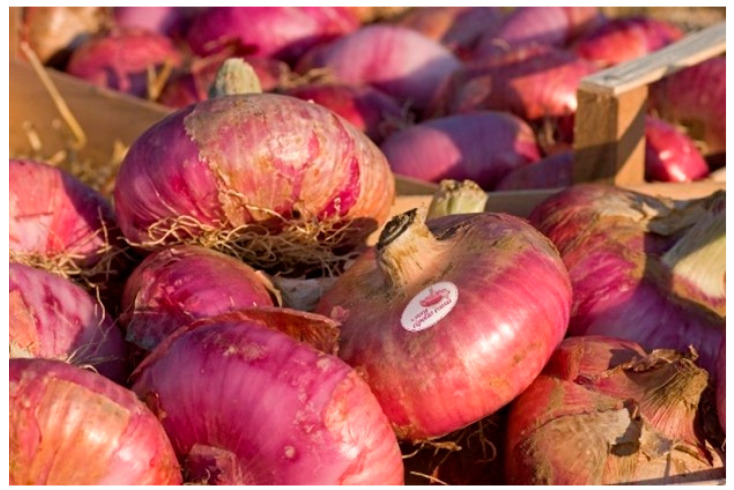
Bulbs of the “Cipolla di Acquaviva” landrace.

The landrace known as “Cipolla rossa di Acquaviva” (Acquaviva red onion, further referred to as ARO) is cultivated and propagated by local smallholders in the municipality of Acquaviva delle Fonti (Province of Bari, Apulia Region of Southern Italy), an area characterized by freshwater availability and deep well-drained soils. It produces red-colored and flattened bulbs, weighting about 500 g and displaying high solid soluble content and low pungency (Figure 1) [14]. The ARO landrace was originally cultivated as a niche local product; however, its appreciated gustatory profile, recently resulting in the obtainment of the “Slow Food Presidium” quality mark, led to a recent sudden rise of its market value and thus cultivation area. This might have ultimately caused the intentional or unintentional contamination of bulbs intended for crop propagation with foreign germplasm. Possible contamination might have occurred with the gene pool including the onion landrace “Cipolla ramata di Montoro” (further referred to as M), cultivated in the Montorese plain area (Provinces of Avellino and Salerno, Campania Region of Southern Italy). Indeed, the M landrace is phenotypically similar to the ARO landrace but displays a longer shelf life and different soluble solid, anthocyanin, and flavonoid contents [14]. In the framework of a regional project for the safeguard, conservation, and characterization of Apulian germplasm, a preliminary characterization of the ARO landrace was performed, using 11 simple sequence repeat (SSR) markers. This highlighted the occurrence of significant differentiation among ARO populations [14].

Genotyping-by-sequencing (GBS) is a reduced-representation sequencing method allowing to perform low-cost and high-throughput genotyping of crops with large and complex genomes [17,18,19]. Moreover, the use of de novo bioinformatic pipelines, such as UNEAK and Stacks, proved effective in extending the use of GBS to species, such as onion, lacking a reference genome sequence [20,21,22].

Here we describe the application of GBS on onion samples referable to populations commercialized as ARO and M, aiming to investigate the genetic structure of the ARO landrace and provide a basis for its further genetic improvement and traceability.

## 2. Results

### 2.1. GBS Experiment and SNP Calling

Sequencing of a 59-plex GBS library yielded 456 million good barcoded reads. The quality control procedure, including steps to filter variants as well as individuals, resulted in a variant call format (vcf) file containing 5011 SNPs and 53 individuals, which was retained and used for downstream analyses. The filtered vcf file was uploaded and is publicly available at the FigShare repository (https://www.doi.org/10.6084/m9.figshare.20301198, accessed on 4 September 2022).

### 2.2. Population Structure

**Figure 2:**
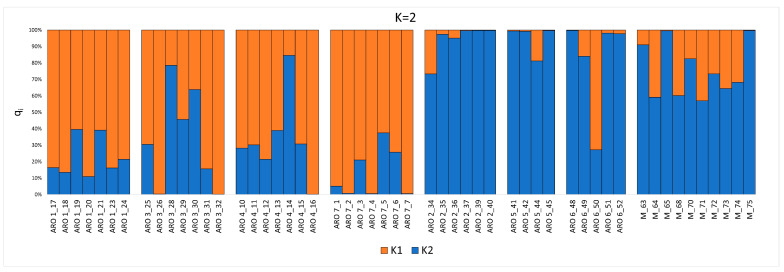
STRUCTURE bar plots referring to 53 onion individuals genotyped in this study for two ancestral subpopulations (K = 2). Each bar refers to an individual and is colored according to the proportion of the genome (q_i_) associated with each K detected.

The genetic structure of the onion germplasm under study was first investigated using the parametric model implemented by STRUCTURE [23]. According to the Evanno’s ΔK test [24], two ancestral subpopulations (K = 2) were assumed to best fit genetic data from the germplasm under study (Figure 2 and Figure S1). With a few exceptions, the K1 ancestry was predominant in individuals belonging to the populations ARO1, ARO3, ARO4, and ARO7, further referred to as the ARO_K1 populations. Conversely, the K2 ancestry was predominant in most individuals of the ARO2, ARO5, and ARO6 populations, further referred to as the ARO_K2 populations, as well as in individuals of the M population.

**Figure 3:**
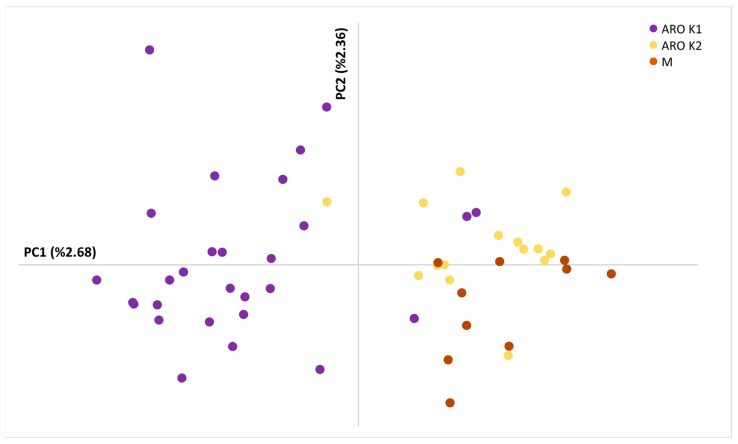
Scatter plot for genetic variation explained by the first two principal components (PCs). Individuals belonging to the ARO_K1, ARO_K2 and M populations are marked with different colors.

Principal component analysis (PCA) was performed as a first non-parametric alternative to study genetic structure (Figure 3). With a few exceptions, the PCA plot based on the first two principal axes (PC1 and PC2) clearly separated individuals belonging to the ARO_K1 population from those belonging to the ARO_K2 and M populations, which fell in different quadrants.

**Figure 4:**
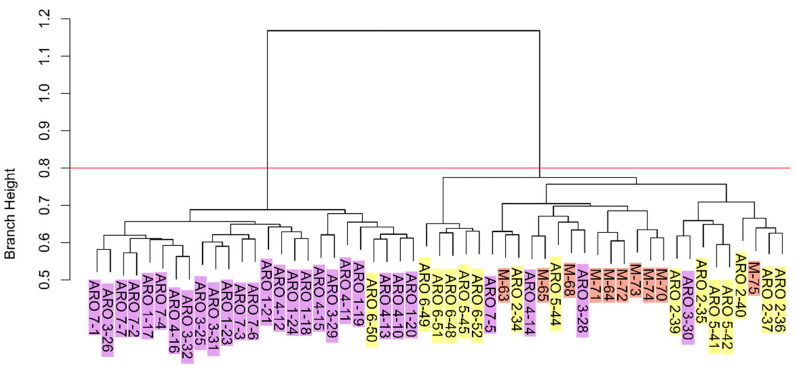
Hierarchical clustering. Labels at the leaves of the dendrogram indicate the population ID (ARO1-7 and M) and the individual ID number. Purple, yellow, and orange label highlighting indicate sampling of individuals within the ARO_K1, ARO_K2 and M populations, respectively. The red line cuts the dendrogram for two major genetic clusters.

In accordance with the results mentioned above, hierarchical clustering highlighted the occurrence of two major clusters, one mainly referable to ARO_K1 individuals, and the other to ARO_K2 and M individuals (further referred to as the ARO_K1 and ARO_K2/M clusters, respectively) (Figure 4). Exceptions included four individuals belonging to the ARO_K1 populations (one from the ARO4 and ARO7 populations, and two from the ARO3 population) that grouped in the ARO_K2/M cluster. ARO1 was the only ARO_K1 population whose individuals all grouped in the ARO_K1 cluster.

Pairwise estimates of the Wright’s F_ST_ statistics ranged from 0 (ARO3 vs. ARO1, ARO4 vs. ARO3, and ARO7 vs. ARO1) to 0.113 (ARO5 vs. ARO7) (Table 1). Permutation analysis on F_ST_ estimates indicated, for the p = 0.05 threshold, non-significant differences among the ARO_K1 populations, and significant differences between any of the ARO_K1 populations and the M population (Table 1). Notably, in accordance with genetic structure analyses, no significant difference was found between any of the ARO_K2 populations and the M population (Table 1).

Population genetic analysis showed a higher number of alleles (Na), the number of effective alleles (Ne), the Shannon’s information index (I) and the observed heterozygosity (Ho) for the ARO_K1 populations, indicating higher genetic diversity. The observed heterozygosity (Ho) was close to the expected heterozygosity (He) for the ARO_K1 populations, thus determining a low fixation index (F), ranging from 0.022 to 0.057. On the contrary, the F values obtained for the ARO_K2 and M populations were considerably higher, ranging from 0.145 to 0.279 (Table 2).

### 2.3. Private Allele Identification

Aiming to provide a tool for product traceability, a search was performed to identify alleles that distinguish the ARO_K1 populations from the M population. In total, 596 private alleles were identified. Private alleles occurred in most cases at low frequency (Figure S2); however, 25 were highly discriminant, as they occurred with a frequency higher than 0.5 and up to 0.65 (Table S1).

## 3. Discussion

The concept of landrace has been debated for a long time by the scientific community [25]. Zeven [26] was the first author to consider landraces as dynamic plant populations, which may evolve through contamination from allochthonous genetic material. However, as stressed by the same author, such a contamination should involve “a few individuals”, and therefore be of limited extent, in order to preserve two key attributes contributing to the formal definition of a landrace, i.e., long history of cultivation in a specific area and recognizable phenotypic identity [27]. Here, we performed a fine-scale genetic study to test the hypothesis that the recent rise of the ARO market value, and thus the ARO cultivation area, resulted in significant contamination with foreign germplasm, which altered the original genetic structure of this landrace. Such contamination was mostly expected from the gene pool of the widespread allochthonous M landrace, which displays a similar bulb appearance but a longer shelf life, and therefore would allow higher income when marketed as ARO. Both parametric and non-parametric genetic structure analyses indicated genetic contamination of germplasm commercialized as ARO with a gene pool including the M landrace, as several ARO individuals, especially from the ARO2, ARO5 and ARO6 populations, were genetically closer to M than other ARO individuals (Figure 2, Figure 3 and Figure 4). Out of seven populations marketed as ARO, each one propagated by a different smallholder, there were three, named ARO_K2 populations after STRUCTURE analysis, that were not significantly different from the M population. The remaining four populations considered in our study, collectively named ARO_K1 populations, formed a distinct group, and contained a population (ARO1) that, based on interviews with farmers, was indicated for sure as directly descendant from germplasm cultivated in the municipality of Acquaviva delle Fonti during the 1980s. Based on this body of evidence, we speculate that the ARO_K1 populations indeed reflect the original genetic structure of the ARO landrace.

The reduction of agronomic performance due to inbreeding (i.e., mating among closely related individuals), referred to as inbreeding depression, strongly limits genetic gains in outcrossing crop species, including onion [28,29,30]. Here, based on the estimation of the fixation index parameter (F), we found that negligible inbreeding gave rise to the ARO_K1 populations (0.022 ≤ F ≤ 0.057), whereas significant inbreeding contributed to the ARO_K2 populations (0.145 ≤ F ≤ 0.279) and the M population (F = 0.278). This result, which likely reflects a larger number of individuals used to propagate the ARO_K1 populations, has two major implications: (i) selection within the ARO_K1 populations might lead to phenotypic improvement without causing obvious deleterious phenotypic effects due to inbreeding depression; (ii) the ARO_K2 and the M populations might possibly already suffer from inbreeding depression, which could be exacerbated by further within-population selection activities. Higher chance of success in performing selection on the ARO_K1 populations derives from their higher genetic diversity, as revealed by the number of alleles (Na), the number of effective alleles (Ne), the Shannon’s index statistics, and the observed heterozygosity (Ho) statistics (Table 2).

Genetic traceability of landraces is of great value for the valorization of typical productions, as well as their protection from frauds [31]. Here, the analysis of about 5000 polymorphic loci identified by GBS allowed the identification of several private alleles for the ARO_K1 populations, which contributed, together with loci with different allele frequency, to the genetic differentiation between the ARO_K1 and M populations (Table 1). Most of the private alleles occurred at low frequency, in accordance with the notion that most SNPs identified by GBS have a low minor allele frequency [18]. Nonetheless, 25 alleles occurred at high frequency (*p* ≥ 0.5), suggesting the possibility to implement traceability tests for the ARO landrace. In general, the results of our study indicate the possibility to exploit SNP variation to develop molecular barcodes for onion landrace traceability or protection. This might be facilitated by the availability of user-friendly technologies for high-throughput SNP detection [32,33,34], together with recent advances in onion genomics [35].

Overall, this study clearly indicates the occurrence of genetic structure in germplasm marketed as ARO, which likely arose from genetic contamination of the original landrace. In addition, it delivers valuable information for breeding activities to be performed on the ARO landrace, and actions to be addressed for its valorization and protection by means of molecular tools.

## 4. Materials and Methods

### 4.1. Plant Material

**Figure 5:**
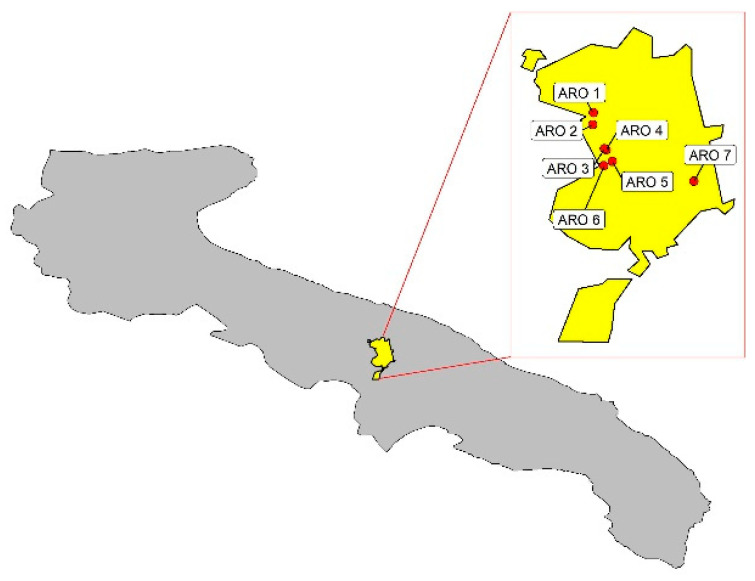
Localization of the different ARO populations genotyped in this study within the municipality borders of Acquaviva delle Fonti (Apulia Region, Southern Italy).

A collection of 59 individuals was analyzed in this study. Of these, 49 represent seven onion ARO populations, each one collected from a different smallholder operating in the municipality of Acquaviva delle Fonti (Figure 5), and 10 were obtained from the Committee for the promotion of the M landrace (https://www.cipollaramatadimontoro.it/index.php, accessed on 4 September 2022). Based on interviews with smallholders, the population referred to as ARO1 was indicated to certainly descend from germplasm cultivated in the municipality of Acquaviva during the 1980s. Plants were grown under the same environmental conditions at the experimental farm “P. Martucci” of the University of Bari “Aldo Moro” (41°1′22.08″ N, 16°54′25.95″ E).

### 4.2. GBS Assay and SNP Filtering

Young leaves were sampled and stored at −80 °C until use. Leaves were washed three times with the STE buffer (0.25 M sucrose, 0.03 M Tris, 0.05 M EDTA) [36] to remove polysaccharides, then total genomic DNA was extracted according to the protocol described by [37]. DNA quality and concentration were checked by the Qubit fluorometer (Illumina ThermoFisher Scientific, Waltham, MA, USA), and about 2 μg of DNA for each sample were used for the GBS assay. This was conducted as described by [17], using the *Ape*KI restriction enzyme to prepare the DNA library and the Illumina HiSeq 2500 system (paired ends) for sequencing.

To perform SNP call and generate a variant call format (vcf) file, the UNEAK pipeline [21] in TASSEL 3 [38] was used, as no onion reference sequence was available. The quality control procedure filtered out SNPs with a call rate < 60% and a minor allele frequency (MAF) < 5%, and individuals with a call rate < 50%.

#### 4.2.1. Population Structure and Genetic Relationships among Individuals

Parametric analysis of population structure was performed with the software STRUCTURE (v.2.3.4) [23], considering 1 to 10 hypothetical subpopulations (the K parameter) with 10 independent runs for each K, a burn-in period of 25,000, and 100,000 Markov chain Monte Carlo iterations. The best K value was inferred by the calculation of the ΔK statistics [24] using the software Structure Harvester [39]. Non-parametric study of the genetic structure was performed by principal component analysis (PCA), which was carried out in TASSEL 5 [38], based on the pairwise identity by state (IBS) distance. In addition, the AWclust package [40] was used to investigate relationships among individuals, based on the allele sharing distance matrix (ASD) and the Ward’s minimum variance clustering algorithm.

Genalex 6.5. [41] was also used to compute pairwise Wright’s F_ST_ estimates among populations and to test the null hypothesis of no significant difference based on 999 permutations. In addition, the same software could compute several diversity indexes (number of alleles (Na), number of effective alleles (Ne), observed heterozygosity (Ho), expected heterozygosity (He), Shannon’s information index (I)), and the inbreeding coefficient (F) associated with each population.

#### 4.2.2. Identification of Private Alleles

Genalex 6.5. was used to search for private alleles, which only occurred in the set of four ARO populations whose ancestry was mostly referable to the cluster K1 identified by STRUCTURE analysis. In addition, the same software could derive the frequency associated with each private allele.

## Figures and Tables

**Table 1:** Pairwise Population F_ST_ values (below diagonal). Probability, P (rand ≥ data) based on 999 permutations, is shown above diagonal.

		ARO_K1				ARO_K2			
		ARO1	ARO3	ARO4	ARO7	ARO2	ARO5	ARO6	M
**ARO_K1**	**ARO1**	0	0.509	0.470	0.433	0.001	0.001	0.012	0.012
	**ARO3**	0	0	0.497	0.421	0.003	0.009	0.045	0.030
	**ARO4**	0.001	0	0	0.319	0.001	0.006	0.070	0.037
	**ARO7**	0	0.002	0.006	0	0.001	0.001	0.002	0.002
**ARO_K2**	**ARO2**	0.078	0.061	0.062	0.093	0	0.464	0.316	0.082
	**ARO5**	0.094	0.073	0.077	0.113	0.003	0	0.438	0.161
	**ARO6**	0.048	0.036	0.033	0.063	0.010	0.011	0	0.279
	**M**	0.030	0.022	0.022	0.042	0.019	0.021	0.009	0

**Table 2:** Statistics on the onion populations genotyped in this study.

	CODE	Na	Ne	I	Ho	He	F
**ARO_K1**	ARO1	1.911	1.619	0.518	0.33	0.352	0.056
	ARO3	1.904	1.61	0.513	0.327	0.349	0.054
	ARO4	1.891	1.615	0.511	0.325	0.348	0.057
	ARO7	1.905	1.617	0.515	0.343	0.351	0.022
**ARO_K2**	ARO2	1.69	1.469	0.399	0.181	0.272	0.279
	ARO5	1.481	1.349	0.311	0.163	0.215	0.19
	ARO6	1.696	1.481	0.406	0.225	0.277	0.145
	M	1.88	1.578	0.49	0.23	0.332	0.278
	Mean	1.795	1.542	0.458	0.265	0.312	0.126

## Data Availability

Data are available at the FigShare repository (doi:10.6084/m9.figshare. 20301198).

## Supplementary Materials

The following supporting information can be downloaded at: https://www.mdpi.com/article/10.3390/plants11182388/s1. Figure S1: Evanno’s ΔK plot associated with STRUCTURE genetic analysis. Figure S2: Frequency distribution of the 596 private alleles distinguishing the ARO K1 population from the M population. Red bins highlight the distribution of private alleles with a frequency above 0.5. Table S1: Private alleles distinguishing the ARO_K1 populations from the M population. Only alleles with a frequency above 0.5 are shown. Information on the SNP locus name, nucleotide, and frequency are reported.
