# Supplementary material for: Genotyping-by-Sequencing Defines Genetic Structure within the “Acquaviva” Red Onion Landrace
Source: Plants (Basel). 2022 Sep 13;11(18):2388. doi: 10.3390/plants11182388 (PMC9502971; doi:10.3390/plants11182388)
Supplement: Supplementary file 1 [file plants-11-02388-s001.zip › plants-1889995-supplementary.pdf]

plants

MDPI

Supplementary Materials

Genotyping-By-Sequencing Defines Genetic Structure within The "Acquaviva" Red Onion Landrace

1. Supplementary Data

The vcf file analyzed in this study was uploaded in the FigShare repository [https://www.doi.org/10.6084/m9.figshare.20301198]

2. Supplementary Figures and Tables

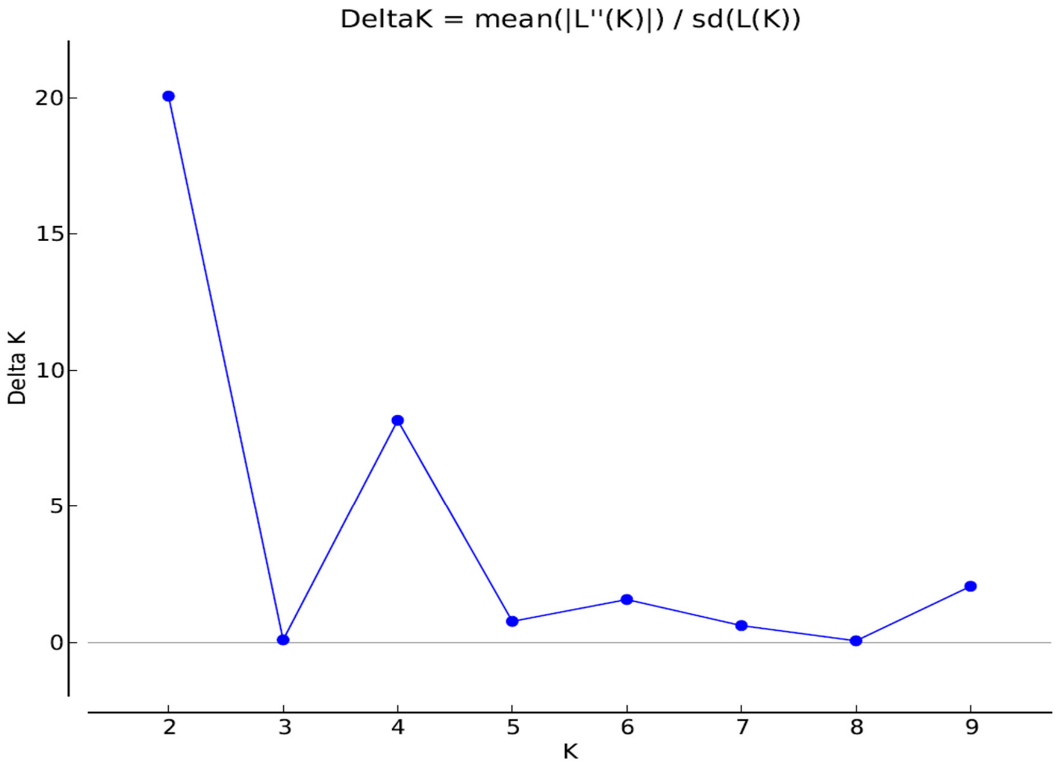

Figure S1. Evanno's ΔK plot associated with STRUCTURE genetic analysis .

2.1. Supplementary Figures

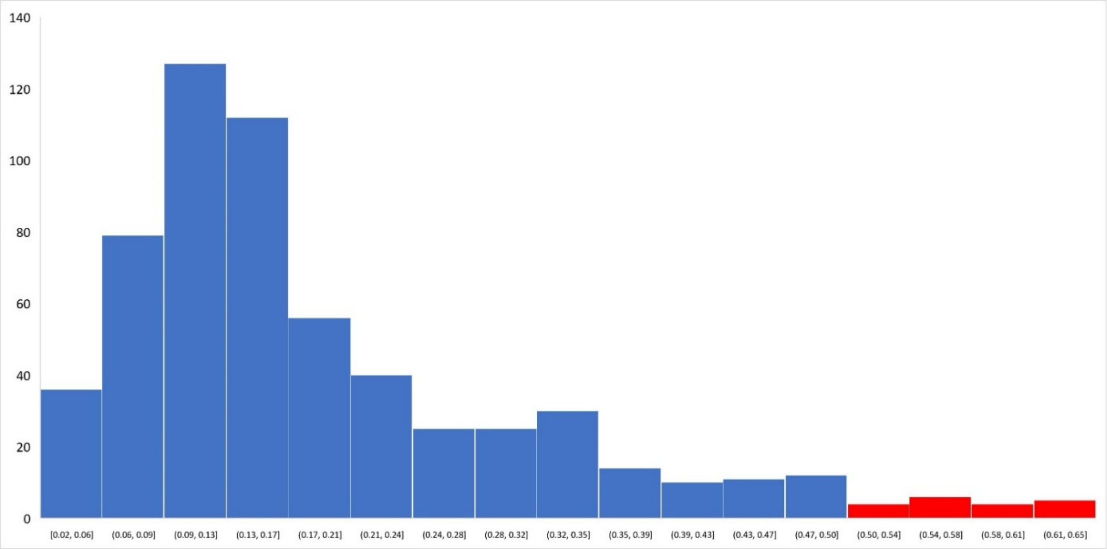

Figure S2. Frequency distribution of the 596 private alleles distinguishing the ARO K1 population from the M population. Red bins highlight the distribution of private alleles with frequency above 0.5.

2.2. Supplementary Table

Table S1. Private alleles distinguishing the ARO_K1 populations from the M population. Only alleles with frequency above 0.5 are shown. Information on the SNP locus name, nucleotide and frequency are reported.

Locus Name	Nucleotide	Frequency
TP261963	A	0.65
TP118650	T	0.64
TP207779	A	0.64
TP167796	A	0.64
TP211157	T	0.63
TP16934	A	0.6
TP307414	C	0.6
TP62851	A	0.6
TP36510	G	0.58
TP105188	C	0.57
TP135073	T	0.57
TP284597	C	0.57
TP286333	A	0.56
TP22187	A	0.55
TP246647	T	0.54
TP151286	T	0.54
TP108000	G	0.53
TP10765	A	0.52
TP25377	G	0.52
TP144838	G	0.5
TP207208	G	0.5
TP227463	C	0.5
TP261711	A	0.5
TP294654	A	0.5
TP6787	A	0.5
